# Content validity of the Japanese version of the health literacy and resiliency scale for youth with chronic illness

**DOI:** 10.3389/fped.2022.978079

**Published:** 2022-11-21

**Authors:** Saya Sekine, Kyoko Kobayashi, Ryota Ochiai, Takashi Higaki

**Affiliations:** ^1^ Department of Child Health Nursing, Graduate School of Nursing Sciences, St. Luke's International University, Tokyo, Japan; ^2^ Department of Nursing, School of Medicine, Yokohama City University, Yokohama, Japan; ^3^ Department of Regional Pediatrics and Perinatology Center for Transition to Adult Congenital Health Disease, Graduate School of Medicine, Ehime University, Ehime, Japan

**Keywords:** transition to adult care, health literacy, resiliency, chronic illness, adolescents, young adults, self-advocacy

## Abstract

Health literacy, which is the ability to find, understand, and use information as well as services to make informed health-related decisions and actions, is essential for ensuring that youths with chronic diseases adapt to and live with their illnesses. However, in Japan, an appropriate approach for measuring health literacy levels among children is yet to be developed. The Health Literacy and Resiliency Scale for Youth (HLRS-Y) was developed by Kathy Bradley-Klug and her colleagues to assess health literacy, resiliency, and self-advocacy/support among youths aged between 13 and 21 years with chronic illnesses in the United States of America (United States). In this study, we aimed to develop a Japanese version of the HLRS-Y and evaluate its content validity. After receiving approvals from the original authors, four nurse researchers with expertise in pediatric nursing translated the scale into Japanese (forward translation). Afterwards, the appropriateness of each expression was examined by a pediatrician. Next, an English native speaker translated the expressions into English (back-translation). We reported the back-translated version of the scale to the original authors to confirm its accuracy. The scale's content validity index (CVI) was evaluated by health professionals working in the fields of pediatric, transitional, and adult health care. The participants rated the items using a four-point scale. Each item was evaluated for a minimum item-level content validity index (I-CVI) value of 0.78. The scale's total and subscale validities were evaluated using a minimum scale-level content validity index based on an average (S-CVI/Ave) value of 0.90. Eleven participants responded to an online survey for evaluating content validity. Of the 36 items, 34 met the I-CVI criteria. Two items did not exceed the criteria's value, but they approximated it. The values of the S-CVI/Ave were 0.96, thereby satisfying the criteria's requirements. Based on the results, it was confirmed that the Japanese version of the HLRS-Y had good content validity. Future studies should examine the factor validity, known group validity, and reliability of this scale.

## Introduction

As the number of youths with special health and medical care needs increases, it is critical for health care providers to maximize the youths' abilities and potential while providing high-quality services appropriate to their developmental stages (1). Achieving this objective requires not only enhancing medical health management but also supporting the development of various abilities, such as health literacy and resiliency.

In 1998, the World Health Organization (WHO) defined health literacy (HL) as “the achievement of a level of knowledge, personal skills, and confidence to take action to improve personal and community health by changing personal lifestyles and living conditions (2).” HL involves the capacity to make sound health decisions, and as one of the health promotion outcomes, it has been attracting attention since the 2000s. HL is a necessary skill for the twenty-first century (3).

Research on HL has developed significantly over the past two decades. Prior studies have shown an association between HL and health outcomes among healthy adults. Low levels of HL are associated with poor health knowledge, noncompliance with medical visit schedules, low rates of medication verification when taking medications, and high rates of hospitalization as well as emergency room usage (4). The acquirement and promotion of HL also results in increased access to adequate health services and health management as well as improved healthcare provider-patient relationships, health-related decisions, and lifestyle choices (5–7).

On the other hand, research on HL among children has been limited owing to the lack of appropriate measurement tools. Although several scales for assessing fundamental HL among children exist, there is no self-administered scale focusing on the independent and subjective evaluation of HL in specific conditions (8). Further research on HL scales that consider various demographic characteristics, such as age, disease patterns, family relationships, social connections, and other factors, is required to ensure that children are respected (9, 10).

In 2017, Kathy Bradley-Klug et al. developed the Health Literacy and Resiliency Scale for Youth (HLRS-Y) to assess HL, resiliency, and self-advocacy/support among 13 to 21-year-olds suffering from chronic ilnesses in the United States (11, 12). The researchers who developed the original scale determined that self-care/management, adaptive coping, and a supportive environment are essential for ensuring that children with chronic illnesses adapt and survive (11, 12). To achieve this objective, they designed the scale to focus on HL (the ability for an individual to make daily decisions regarding their health care and assert their health-related needs) and resilience (self-efficacy, self-regulation, and a sense of connectedness) as essential components aimed at helping children suffering from chronic illnesses adapt and grow (11, 12).

The target population for this scale was defined as youth aged between 13 and 21 years. The items included in the scale were designed to consider the complex and specific situations associated with prioritizing autonomy, building relationships with other individuals outside the family, and integrating identity when dealing with chronic health conditions. This scale comprised 36 items: 10 for HL, 12 for resilience, and 14 for self-advocacy/support, which were rated using a four-point scale (strongly agree, agree, disagree, and strongly disagree). The scale has been verified for factor validity and the Cronbach's alpha coefficients for each subscale were 0.87 for HL, 0.93 for resilience, and 0.79 for support and self-advocacy (11, 12). The strength of this scale is that it is self-administered, and it questions participants about independent health management factors that are necessary for them to live with chronic illnesses without giving them the feeling that they are being tested.

However, currently, in Japan, HL levels among children and adolescents suffering from chronic illnesses are not known, and an appropriate approach for measuring HL levels among such individuals is yet to be developed. Therefore, it was necessary to translate the HLRS-Y into Japanese. Referring to the consensus-based standards for the selection of health status measurement instruments (COSMIN) guidelines (13), first, permission to use and translate the scale was obtained from the authors of the original version. Second, forward translation of the scale was independently performed by four nurse researchers, fluent in English and specializing in pediatric nursing. Afterwards, the contents were reviewed and summarized through discussion. Next, the appropriateness of each expression was examined by one pediatrician. Third, the back-translation into English was performed by an English native speaker. Finally, it was verified whether the Japanese expressions were smooth and easy to understand among youths between the ages of 13 and 21 while still remaining as direct a translation as possible.

Therefore, in this study, we aimed to develop the Japanese version of the HLRS-Y and evaluate its content validity.

## Material and methods

### Content validity index survey

#### Participants

Through snowball sampling, 18 experts in the fields of pediatric, transitional, and adult health care were requested to participate in this study. The experts included physicians, nurses, university professors in the fields of nursing and education, social workers, psychologists, and independence supporters for specific pediatric chronic disease. We also recruited adult patients with congenital heart diseases as well as survivors of childhood cancer.

#### Survey method

The survey was conducted using a web-based self-administered questionnaire. The survey period was February 16, 2022 to February 25, 2022.

#### Survey contents

To obtain the professional background of the participants, we asked them about their current role, qualifications, and years of experience engaging with patients aged between 13and 21 years with childhood-onset diseases (this included the participants' years of clinical experience, education, and research).

Regarding the content validity of the Japanese version of the HLRS-Y, we asked the participants to rank the validity of the items in each domain (HL, resiliency, and self-advocacy/support) on a four-point Likert scale: 4 = highly relevant; 3 = quite relevant; 2 = somewhat relevant; and 1 = not relevant. Additionally, for each domain, we asked the participants to provide their revisions and comments regarding the items in the free-description section.

#### Analysis method

We relied on the item-level content validity index (I-CVI), which is calculated using the number of respondents providing a rating of three or four divided by the total number of respondents for all the items. The criterion value for I-CVI was set at 0.78 (14). We also evaluated the scale-level content validity index based on average (S-CVI/Ave), which is the average of the I-CVI. The criterion value for S-CVI/Ave was set at 0.90 (14).

#### Ethical considerations

In compliance with the Declaration of Helsinki, 1964, we considered the protection of human rights (15). The human rights of the individuals were defended. In this study, there was no direct intervention on the participants, and it does not contain any personal information. It was also explained to the participants that they could choose to respond and there would be no disadvantages associated with non-participation.

## Result

### Demographics of the participants

Eleven valid responses were received (valid response rate: 61.1%). The eleven participants were as follows: two specialist physicians (pediatric oncologist and adult cardiologist), one Certified Nurse Specialist in pediatric nursing, one social worker, one university professor of nursing, one university professor of education, one psychologist, one independence supporter for specific pediatric chronic disease, and three patients (one pediatric cancer survivor and two patients with congenital heart disease). The mean level of the years of clinical experience and years of education as well as research involving individuals suffering from chronic illnesses who are aged between 13 and 21 years was 21.0 (SD = 12.5).

### CVI

Regarding the scale's total validity, the value of the S-CVI/Ave was 0.96, which exceeded the criterion's expected levels (0.90). As shown in Table 1, 26 out of 36 items had an I-CVI of 1.00, which is the upper limit. The other four items scored 0.91, each with another four items with a score of 0.82.

**Table 1:** Content validity Index of HLRS-Y Japanese version.

			I-CVI			
Subscale	No.		Total (*n* = 11)	Medical expert (*n* = 4)	Psychosocial expert (*n* = 4)	Patient (*n* = 3)
Health Literacy	H1	I know the common symptoms of my illness.	1.00	1.00	1.00	1.00
	H2	I can tell if my symptoms are serious or not.	1.00	1.00	1.00	1.00
	H3	I understand my illness well.	1.00	1.00	1.00	1.00
	H4	I know the medicine I need for my illness.	1.00	1.00	1.00	1.00
	H5	I know the correct amount of medicine for me.	0.73	0.75	0.75	0.67
	H6	I know what kind of exercise or activities are not good for my health.	1.00	1.00	1.00	1.00
	H7	I know when to tell friends and family about things I can't do and things I should be aware of.	1.00	1.00	1.00	1.00
	H8	I understand that illness may affect school and workplace activities in various ways.	0.91	1.00	0.75	1.00
	H9	I understand that illness may affect my relationships with friends in various ways.	0.73	0.75	0.50	1.00
	H10	I am learning about my illness while talking with people who have had the same experience as me.	1.00	1.00	1.00	1.00
Resiliency	R1	I am optimistic about my future.	1.00	1.00	1.00	1.00
	R2	I accept my illness as one of my characteristics.	1.00	1.00	1.00	1.00
	R3	I think about how best to spend my time as I live with my illness.	1.00	1.00	1.00	1.00
	R4	I think positively or humorously about things even in difficult times.	1.00	1.00	1.00	1.00
	R5	I try to be optimistic about life.	0.82	0.75	0.75	1.00
	R6	I think about ways to enjoy playing with friends and family while living with illness.	1.00	1.00	1.00	1.00
	R7	I think the experience of illness will somehow be useful in the future.	1.00	1.00	1.00	1.00
	R8	If you look at someone who is living well with a similar illness, it can be helpful to your own methods of living with illness.	1.00	1.00	1.00	1.00
	R9	I am relieved when I meet or talk with people my age or older who are living with illness.	0.82	1.00	0.50	1.00
	R10	The people around me can help me to laugh about my illness.	0.91	0.75	1.00	1.00
	R11	Since the people around me enable me to participate in events and activities, I am able to have the same experiences as everyone else.	1.00	1.00	1.00	1.00
	R12	Being able to talk to someone about my own experience helps me to live with my illness.	1.00	1.00	1.00	1.00
Support/Self Adovocacy	S1	I understand that illness may affect my parents and other people in various ways.	0.83	0.75	0.75	1.00
	S2	Teachers at school know about my illness.	1.00	1.00	1.00	1.00
	S3	I tell people around me when I'm feeling unwell.	1.00	1.00	1.00	1.00
	S4	I am learning about my disease from medical professionals.	1.00	1.00	1.00	1.00
	S5	I limit and adjust daily activities according to my condition.	1.00	1.00	1.00	1.00
	S6	I consider my physical condition and when necessary, take breaks or take it easy more than usual.	0.91	1.00	1.00	0.67
	S7	I seek the help of family and friends to live with my illness.	1.00	1.00	1.00	1.00
	S8	There are people around me who take care of me.	1.00	1.00	1.00	1.00
	S9	I seek the help of my school teachers to live with my illness.	1.00	1.00	1.00	1.00
	S10	My parents have learned much about my illness and help me live with it.	1.00	1.00	1.00	1.00
	S11	There is always someone who cares about whether or not I need help, due to my illness.	0.82	1.00	0.75	0.67
	S12	I have family and friends who I can rely on when I have to go to hospital, stay in hospital, go for tests or undergo surgery.	1.00	1.00	1.00	1.00
	S13	I am receiving the necessary consideration to be successful in my school life.	1.00	1.00	1.00	1.00
	S14	The teachers at school understand the necessities regarding my physical condition.	0.91	1.00	1.00	0.67
S-CVI/Ave			0.96	0.97	0.94	0.94

I-CVI: item-level content validity index.

S-CVI/Ave: scale-level content validity index based on an average.

Medical expert includes physicians, nurse, and university professor in nursing.

Psychosocial expert includes university professor in education, social worker, psychologist, and independence supporter for specific pediatric chronic disease.

There were two items for which the I-CVI values were 0.73, which was less than the criterion's expected levels (0.78). Both of these items (H5 and H9) were related to HL. Regarding H5, “I know the correct amount of medicine for me,” the participants who provided ratings of less than three or four were an adult cardiologist, an independence supporter for specific pediatric chronic disease, and a patient with congenital heart disease. The cardiologist commented that “I think the dosage for medication (s) is unnecessary knowledge.” Regarding H9, “I understand that illness may affect my relationships with friends in various ways,” the participants who provided ratings of less than three or four were a pediatric oncologist, a university professor of education, and a psychologist. The psychologist commented that “It is difficult to answer because the question is not clear.”

The I-CVI was above the criterion's expected levels. However, one patient provided a comment regarding R10: “The people around me can help me to laugh about my illness.,” stating that the phrase “laugh about my illness” might have a negative connotation in Japanese. For any other items, all the experts did not provide any comments regarding the invasive nature of the expressions to the patients involved in this study.

For some items in the self-advocacy/support criterion, there were comments pointing out that the word “school” might have resulted in difficulty in providing responses because the target population's age was up to 21 years. Additionally, a Certified Nurse Specialist commented, “Not everyone needs support. It is important to be able to ask for support if needed.”

## Discussion

In this study, we developed a Japanese version of the HLRS-Y, and its content validity was assessed by eleven experts involved in supporting children with chronic illnesses, including the patients themselves. Previous studies recommend the participation of at least five experts in the CVI evaluation (16). The COSMIN Guidelines also recommend the participation of patients during content validity assessments (13). This study meets these requirements and is considered to have achieved a certain level of content validity evaluation. The results of this study were as follows: (1) The CVI of the items of HLRS-Y were above the criterion value, except for two items; (2) Experts, including the patients involved in this study confirmed that the items were not invasive; (3) Some items, such as school-related ones, might require attention in the way the questions were constructed to avoid the inclusion of situations that might not be applicable to some participants. These findings are discussed in detail as follows:

(1) **The CVI of the items of HLRS-Y were above the criterion value, except for two items.**

Overall, the value of the S-CVI/Ave of the HLRS-Y was 0.96, and 34 out of the 36 items met the I-CVI criteria, thereby demonstrating that the scale has good overall content validity. However, two items (H5, H9) did not meet the expected criterion's values. Regarding H5, it could be assumed that H5 did not exceed the criterion values because the experts tended to believe that the importance of medication management was in the name, timing, and effect of the medication rather than in the dosage. Several previous studies suggest that healthcare providers should consider various aspects to ensure that children suffering from chronic illnesses adhere to appropriate medication behaviors (e.g., providing information regarding the type, dosage, duration of use, and common side effects of medications, easy-to-understand medication regimens, creating self-administration plans, and regular consultations with physicians) (17–19). The Transition Readiness Assessment Questionnaire (23 items in total), which is one of the world's most used measures of transition readiness among patients with childhood-onset diseases, includes six items related to adverse medication reactions, appropriate intake, and drug names and dosages (20, 21). Because the HLRS-Y includes more items related to daily life, it has fewer items related to medical care than the TRAQ, with only two items related to medications. Because one of the items (H5) was related to medication dosage, the experts may have thought that there were other items required, such as the names and adverse reactions. On the other hand, the physician commented, “I believe specialized knowledge is unnecessary for understanding health.” Additionally, the patients stated, “I believe it depends on the individual,” and “As a patient, I believe I understand the bottom line, but it is still not as good as relying on that of the medical professionals.” We decided not to modify H5 in the Japanese version of the HLRS-Y because the scale focuses on daily life in general, and several participants commented that detailed knowledge was not always essential.

Regarding H9, the psychologist pointed out that it was difficult to understand the purpose of this question. In this item, “my health impacts” is assumed to be, for example, that the illness causes limitations in behavior, thereby making it difficult to move at the same pace as a group of friends. However, it could be difficult to understand from the first reading. Although children suffering from chronic illnesses are at a higher risk for emotional and behavioral problems (22), good friendships have been shown to have a positive impact on their psychological and physical health (23). In the Japanese version of the HLRS-Y, H9 was not modified in terms of prioritizing comparability with the original scale.

(2) **Experts, including patients, confirmed that the items were not invasive.**

In this study, the experts did not comment on the invasiveness of the expressions of all the items, except for R10. Regarding R10, one patient pointed out that the expression “laugh about my illness” may have a negative meaning. Therefore, we changed the expression from “humor” to “cheerful,” and we modified the item as follows: “People around me are cheerful and help me with my illness.”

(3) **Some items, such as school-related ones, might require some attention in the way the questions were constructed to avoid including situations that might not be applicable to some participants.**

There were items that asked about the situations in school. Because the target population for this scale was up to the age of 21 years, some participants might have already graduated from high school and were employed. The school situation was not applicable for them. In fact, patients of the age at which they are eligible for HLRS-Y may not have attended school. Specifically, severely diagnosed patients are reported to have a lower educational background (24, 25), and the more severely diagnosed patients are, the more likely for them to be out of school. However, this also suggests that severely ill patients experience some difficulties in school. Therefore, in the Japanese version of the HLRS-Y, we decided to include items related to school life. However, we shall consider adding “not applicable” as a response option.

### Limitation

The expert panel included only three patients (pediatric cancer survivor and patients with congenital heart disease). When the original version of the HLRS-Y was developed, a wide range of patients was included in the study participants, including those with diabetes and juvenile rheumatoid arthritis (11). Therefore, it is necessary to increase the number of patients and the types of diseases. Guardians should also be included because they are likely to be concerned about whether the scale is invasive for their children.

In conclusion, the Japanese version of the HLRS-Y was found to have good content validity. Future studies should examine the factor validity, known group validity, and reliability of this scale.

## Data Availability

The original contributions presented in the study are included in the article/Supplementary Material, further inquiries can be directed to the corresponding author/s.
